# Weak Local Adaptation to Climate in Seedlings of a Deciduous Conifer Suggests Limited Benefits and Risks of Assisted Gene Flow

**DOI:** 10.1111/eva.70001

**Published:** 2024-09-15

**Authors:** Beth Roskilly, Sally Aitken

**Affiliations:** ^1^ Forest and Conservation Sciences University of British Columbia Vancouver British Columbia Canada; ^2^ USDA‐Forest Service Pacific Northwest Research Station Corvallis Oregon USA

**Keywords:** assisted migration, climate change, cold hardiness, ecological genetics, local adaptation, phenology

## Abstract

Assisted migration provides a potential solution to mitigate the increasing risks of forest maladaptation under climate change. Western larch (*Larix occidentalis* Nutt.) is a deciduous conifer species undergoing assisted migration beyond its natural range in British Columbia into areas that have become suitable based on climatic niche modelling. We established a seedling common garden experiment in raised beds in a warm location outside the natural range for three growing seasons, with 52 natural populations from across the species range and 28 selectively bred families from British Columbia. Intraspecific genetic variation in growth, phenology and cold hardiness was analyzed to test for signals of local adaptation and the effects of selective breeding to better understand the implications for assisted migration and breeding for future climates. We found weak differentiation among populations in all traits, with the proportion of additive genetic variance (*Q*_ST_) ranging from 0.10 to 0.28. Cold hardiness had the weakest population differentiation and exhibited no clines with geographic or climatic variables. Selective breeding for faster growth has maintained genetic variation in bud flush phenology and cold hardiness despite delaying bud set. The weak signals of local adaptation we found in western larch seedlings highlights that assisted gene flow among populations is likely to have limited benefits and risks for mitigating maladaptation with climate change. Our findings suggest that assisted migration outside of the range and selective breeding may be important management strategies for western larch for future climates.

## 1 Introduction

Forest trees are becoming increasingly vulnerable to climatic stress with rapid climate change (Allen et al. 2015; Hartmann et al. 2022). Shifting climates and greater climatic variation are disrupting historical patterns of local adaptation in tree populations, increasing the risk of maladaptation and the cascading ecosystem‐level consequences of declining forest health, and presenting new and urgent challenges for forest management (Millar and Stephenson 2015). A potential management solution for mitigating the increasing risk of maladaptation is assisted migration, the managed translocation of genetic material to areas where it is forecasted to be optimally adapted in the future to facilitate more rapid adaptation to climate change (Aitken and Whitlock 2013). Assisted migration can include the movement of genetic material among populations within the current range, that is, assisted gene flow, and assisted migration beyond the current range (Aitken and Whitlock 2013). Tree populations have limited capacity to adapt or migrate quickly enough to track current and future climate change because of their long generation times but they also tend to have high gene flow and limited population isolation, making them well suited for assisted migration.

To evaluate the potential impacts of assisted migration, it is critical to understand patterns of local adaptation to historic climates in trees. More than 250 years of common garden experiments in forestry have shown that local adaptation to climate is common in tree species and that the strength and scale of local adaptation varies among species (Alberto et al. 2013; Leites and Benito Garzón 2023). In common garden studies, population differentiation in relevant traits and the association between traits and source environments is regarded as evidence consistent with local adaptation to climate (Aitken and Bemmels 2016; Wadgymar et al. 2022; Leites and Benito Garzón 2023). Many widely distributed and economically important conifer species exhibit strong signals of local adaptation to climate, while many broadleaf and some conifer species show weak to no signal, suggesting a relatively strong role of phenotypic plasticity to accommodate environmental variation (Leites and Benito Garzón 2023). Phenotypic plasticity is the capacity of a genotype to produce different phenotypes in response to environmental variation. Adaptive phenotypic plasticity can help stabilize fitness traits and the limits of phenotypic plasticity can determine how much environmental change a genotype can withstand before fitness is compromised (Ghalambor et al. 2007). Phenotypic plasticity can buffer populations against environmental change, allowing persistence in the short term, but it can delay genetic adaptation in the long term (Valladares et al. 2014). Understanding the strength and scale of local adaptation relative to phenotypic plasticity in different tree species can help inform effective strategies for assisted migration in future climates.

It is well established that cold temperatures are important drivers of local adaptation in temperate trees, especially conifers, which have a long evolutionary history of adaptation to harsh climatic conditions that has allowed them to dominate temperate and boreal forests throughout the world (Bond 1989). Climate change and assisted migration approaches are likely to shift the timing and distribution of late spring and early fall frost risks relative to the seasonal developmental cycle of locally adapted tree populations (Sang et al. 2021). One of the major challenges of assisted migration for temperate and boreal tree populations is the need to balance the potential risks of frost damage and compromised survival at early seedling stages against the benefit of mature trees optimally adapted to warmer climates decades to centuries later (O'Neill et al. 2014; Erlichman et al. 2024). Further, cold temperatures are important cues for the process of cold hardening in trees in the fall, and warming or more variable temperatures could delay this process resulting in greater vulnerability to cold injury (Bansal et al. 2015). Understanding the potential impacts of climate change and seed transfer on local adaptation to cold temperatures and seasonal frost risks is therefore critical for minimizing risks associated with assisted migration.

Locally adapted temperate and boreal tree populations have evolved timing of active growth so that trade‐offs between growth and survival due to seasonal frost risks are optimized. While trade‐offs between growth and seasonal frost tolerance are well established in natural populations (Howe et al. 2003), trade‐offs or correlated responses associated with selective tree breeding for faster growth are less clear yet the risks associated with compromised frost tolerance are an increasing concern for future tree breeding and assisted migration under rapidly changing climates. Traditional tree breeding programs aim to increase growth gains by selecting for genotypes with faster growth rates, and this may result in an extended growing period. When selection delays growth cessation, there is a risk of a phenological mismatch with seasonal frost risks, resulting in selectively bred trees that are more vulnerable to cold injury compared to natural populations. Alternatively, if selective breeding enhances growth without strong impacts on phenology or cold hardiness, the synchrony with seasonal frost risks could be maintained and seed transfer based on climate could be similar between natural and selectively bred sources. Encouragingly, previous common garden studies of lodgepole pine and interior spruce have found that selective breeding for faster growth has mostly maintained variation in cold hardiness (MacLachlan et al. 2017; MacLachlan, Yeaman, and Aitken 2018).

We studied genetic variation in traits related to the seasonal development cycle of western larch (*Larix occidentalis* Nutt.), a deciduous conifer species undergoing assisted migration trials beyond its current natural range in British Columbia (Rehfeldt and Jaquish 2010; O'Neill et al. 2017). Climate niche modelling has projected that areas of suitable climate for western larch have expanded beyond and contracted within its current range and will continue this trend in the coming decades, making it a desirable candidate for assisted migration beyond the range (Rehfeldt and Jaquish 2010; MacKenzie and Mahony 2021). We established a seedling common garden experiment in raised beds in a location warmer than the natural range for three growing seasons with a range‐wide collection of 52 natural populations to simulate effects of assisted migration and warming. We included 28 selectively bred full‐sib families to compare with natural populations from the same regions to test for the effects of selective breeding on climate‐relevant traits. We measured traits related to the seasonal developmental cycle (height growth, bud phenology, and cold hardiness) to address the following questions: (1) How much population differentiation exists in climate‐relevant traits among natural populations? (2) Does trait differentiation among populations exhibit clinal variation with climatic or geographic gradients? (3) How does selective breeding for faster growth affect variation in climate‐relevant traits?

## 2 Methods

### 2.1 Study Design

**FIGURE 1:**
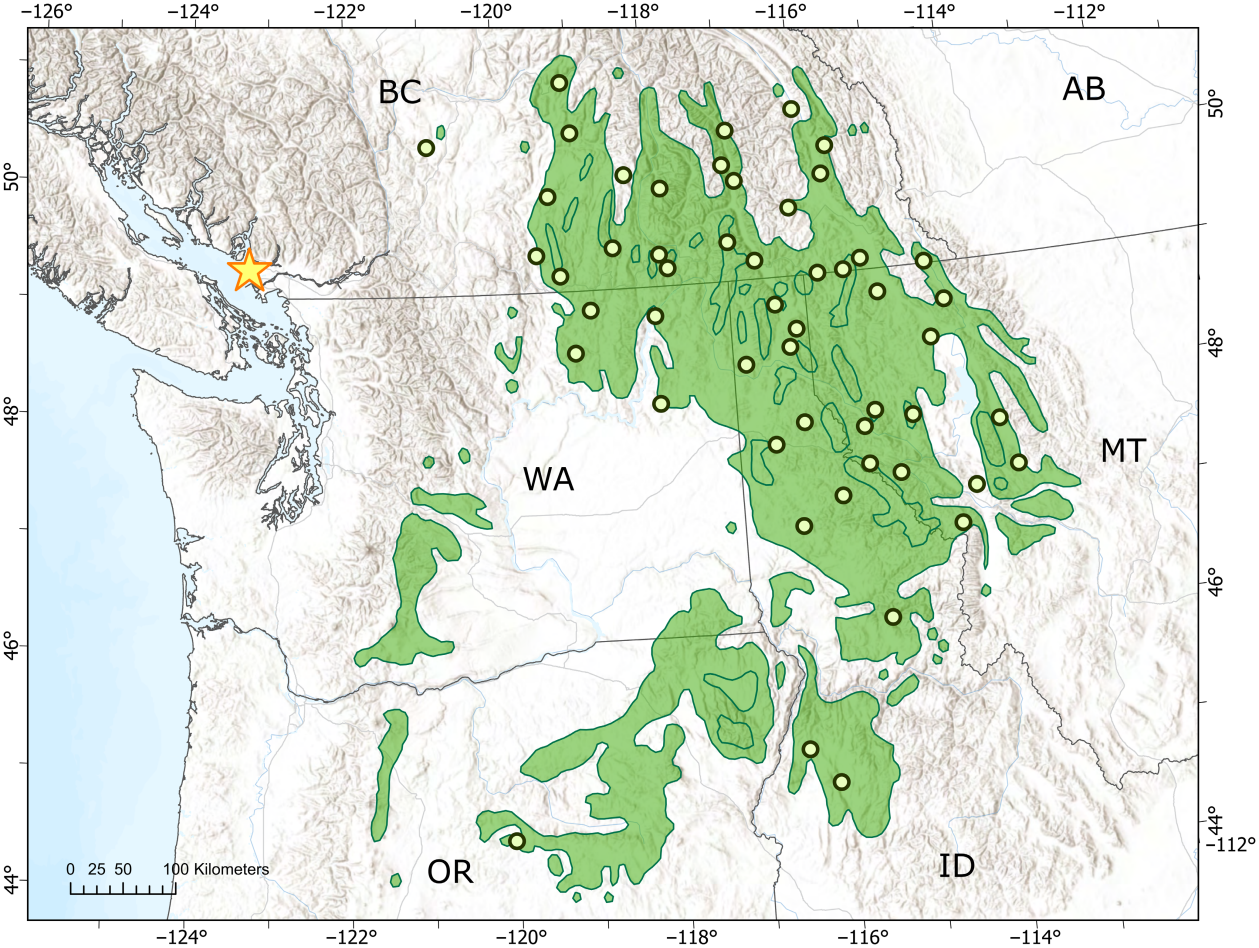
Distribution of 52 sampled natural populations (green circles) and the natural range of western larch in green and location of common garden test site (gold star) in western North America.

We obtained 52 open‐pollinated seedlots from natural populations across most of the natural range (Figure 1). We selected populations from available seedlots dispersed in geography and climate spanning a range of 6° latitude, 7° longitude, 1100 m elevation, 6°C mean coldest month temperature, and 1325 mm mean annual precipitation (Table S1). The common garden site was situated outside of the natural range in Vancouver, BC, which has a warmer climate (Figure S1). Seedlots were obtained from stored collections from the Rocky Mountain Research Station, Inland Empire Tree Improvement Cooperative, and British Columbia Tree Seed Centre. Each seedlot was bulked from at least 10 maternal trees in British Columbia and 5–10 maternal trees in the United States, generally sampled at least 100 m apart at a given site. Populations of the interior portion of the range (i.e., Rocky Mountain region) are well represented but populations from the westernmost portion of the range (i.e., Washington and Oregon) were not included in the study due to the small number of maternal trees sampled per location for the few available seedlots. To test for the effects of selective breeding on traits relative to natural populations, we obtained seed from 28 selected full‐sib families of the western larch breeding program of the British Columbia Ministry of Forests from two breeding zones established in 1990 (Figure S2). Selected full‐sib families included 32 parents total, 28 of which were represented in more than one family, allowing for estimation of additive genetic variation and narrow‐sense heritability values.

Seeds were soaked in water and cold stratified for 3 weeks, then sown in 55 cm^3^ Stuewe & Sons Ray Leach UV containers filled with Sunshine Mix #4 potting media on April 13–16, 2019. Two to four seeds were sown per container depending on expected germination rates of each seedlot and thinned to one seedling after germination. Seedlings were grown in a greenhouse under 18‐h days and an average temperature of 25°C. Trays of 200 seedlings each were kept well‐watered, fertilized with Peters Excel 11‐41‐8 N‐P‐K water soluble fertilizer, and rotated weekly to avoid any spatial effects inside the greenhouse. In order to induce bud set and dormancy, all seedlings were put under a 10‐h photoperiod from September 30th onward. By November 13th, all seedlings had set bud and were moved to controlled chambers set at 4°C in the dark to simulate winter and chill seedlings to break bud dormancy.

We transplanted seedlings to raised outdoor beds in April 2020. Seedlings were planted in four blocks of 240 plants in a complete randomized block design. Seedlings were planted with 4 cm spacing, with three randomly assigned individuals per seed source within each block, 12 plants per seed source, totaling 960 seedlings. Each block was surrounded by a row of buffer seedlings that were not measured to minimize edge effects.

Seedlings were watered through the growing season and we applied Peters Excel 15‐5‐15 N‐P‐K water soluble fertilizer at the beginning of the growing season to avoid nutrient deficiency. Signs of a fungal pathogen infection, identified as *Sirococcus conigenus*, were detected in a small number of seedlings in early June 2021, causing the terminal shoot to curl downwards, the needles to turn brown and eventually fall off. A broad‐spectrum fungicide treatment, Aliette, was administered immediately and plants were regularly monitored for signs of infection. No signs of infection spread were detected after treatment. Seedlings with dead tops resulting from the infection (<5%) were excluded from analyses.

### 2.2 Climate and Geographic Data

We used the geographic coordinates of each population to obtain interpolated climate records for the period of 1961–1990 from ClimateNA (Wang et al. 2016). This is the earliest period that weather stations produced reliable data and was assumed to be early enough to represent historic conditions. For testing phenotype‐environment associations, we selected five annual climate variables based on biological relevance and pruned for collinearity for phenotype‐environment associations: mean coldest month temperature (MCMT), continentality (TD) calculated as the difference between mean warmest month temperature and mean coldest month temperature, frost‐free period (FFP), mean annual precipitation (MAP), and summer‐heat moisture index (SHM). We also analyzed trait associations with geographic variables of latitude, longitude, and elevation.

### 2.3 Trait Data

We measured six traits related to the seasonal development cycle in conifers (height growth components, bud flush, bud set, and fall cold hardiness). Phenotypic data from the third year of growth (2021) was analyzed for this study. Height was measured five times throughout the growing season and final height was measured after growth cessation and final bud set on September 6, 2021. Early growth was estimated as the height on June 21, 2021 (the date seedlings began to set bud for the first time) minus the initial height measured at the beginning of the growing season on May 13, 2021. Early growth includes both determinate growth from shoot primordia that overwintered in buds and indeterminate free growth from neoformed leaves and internodes. In western larch, free growth extends beyond the juvenile stage and remains a significant component of growth potential in mature trees (Schmidt and Shearer 1990). We could not separate determinate and free growth in this study because preformed and neoformed leaves are not readily distinguishable based on morphological differences; therefore, we analyzed early and late season growth instead. Late growth was estimated as final height minus the height on June 21, 2021. Late growth includes free and lammas growth, a form of indeterminate shoot growth resulting from reflushing after initial bud set in the current growing season.

Visual assessments of the timing of terminal bud flush and bud set were collected to analyze the timing of initiation and cessation of height growth, respectively. A binary system was used to record the visual observations. Bud flush was determined as the parting of bud scales and the emergence of needles and bud set as the formation of visible brown bud scales on apical buds. Several seedlings set bud multiple times due to lammas growth, and final bud set was determined as the date after which no reflushing was observed. Observations of terminal bud phenology were recorded every 5 days at the beginning of the growing season until all seedlings had fully flushed from March 8 until April 13, 2021. Bud set was monitored weekly from June 21 to September 22, 2021. Bud flush and bud set dates were analyzed as day of the year starting January 1.

Cold hardiness was measured in October as minimum air temperatures began to approach 0°C at the test site. Measurements involved artificially freezing and measuring electrolytic leakage of branch tissue following a protocol adapted from (Hannerz et al. 1999). Three samples, each comprising three branch segments 5 mm length, were collected from each seedling; two samples were used for two different freeze test temperatures and the third sample was an unfrozen control. Test temperatures were selected based on preliminary freeze tests to result in approximately 50% cold injury. Cold hardiness testing was performed over two consecutive weeks (October 18–28, 2021) to accommodate the large number of samples. Test temperatures were −25°C and −30°C while control samples were placed in a fridge at 4°C. A small amount of silver iodide to nucleate ice crystals and 0.2 mL distilled H_2_O were added to samples before freezing. Samples were exposed to test temperatures for 1 h in a programmable temperature chamber, Tenney model T20C‐3. After freezing, 3 mL of distilled water was added and samples were shaken for 1 h. Electrical conductivity was measured on test and control samples after freezing, and again after heat killing at 95°C in a laboratory oven overnight, using Amber Science Inc. Model 2052 Digital Electrical Conductivity meters. Cold injury was estimated as the percentage of cellular damage using the ratio of electrolytic leakage after freezing relative to total leakage after heat killing at 95°C between test and control samples. The cold injury damage incurred by each seedling at both test temperatures was calculated as a percentage relative to unfrozen control samples using an index of cold injury (*I*) (Flint et al. 1967). We averaged the values of *I* between the two test temperatures and used this mean value for our analyses.

### 2.4 Analyses

### 2.5 Population Phenotypes and Variance Components

We used a linear mixed‐effect model using residual maximum likelihood to estimate best linear unbiased estimates (BLUEs) of natural populations and selected full‐sib families with ASReml‐R version 4.0 (Butler et al. 2018). The following mixed‐effects model was used:

(1) $$Y_{\mathrm{ijk}}=\mu +P_{j}+B_{k}+\epsilon_{\mathrm{ijk}},$$

where $Y_{\mathrm{ij}}$ is the phenotype corresponding to individual *i* from the population *j* and block *k*; μ is the phenotypic global mean across all individuals (fixed intercept); *P* is the coefficient for the fixed effect of the *j*th population; *B* is the random effect of the *k*th block; and $\epsilon_{\mathrm{ijk}}$ is the random residual error term. To account for spatial effects within the experiment, row and column positions within the experiment were incorporated into the random residual term with a correlation structure. Models including the spatially correlated residual term consistently reduced Akaike's information criterion (AIC) values.

We estimated population variance components and standard errors for each trait by fitting mixed‐effects models. The model form used was the same as Equation (1) but with all independent variables set as random effects.

### 2.6 Population Differentiation

We quantified differentiation among populations for each phenotypic trait with two metrics: *V*_POP_, a measure of the amount of phenotypic variance among populations relative to the total phenotypic variance and *Q*_ST_, a standardized measure of the amount of genetic variance among populations relative to the total genetic variance. *V*_POP_ is calculated as the phenotypic variance among populations divided by the sum of the residual variance and the variance among populations. *V*_POP_ is often considered as a proxy for *Q*_ST_ when relationships among individuals within populations are not known. However, it is an underestimation of *Q*_ST_ because in the denominator, the total phenotypic variance is used (i.e., population and residual component), rather than the sum of the among‐population component and twice the additive variance component used to calculate *Q*_ST_ (Liepe et al. 2016). For comparison, we approximated *Q*_ST_ as the phenotypic variance among populations divided by the sum of twice the additive variance component estimated from selected full‐sib families and the phenotypic variance among populations. *Q*_ST_ estimates were then qualitatively compared with *F*_ST_, a standardized measure of neutral genetic differentiation among populations, estimated as global *F*_ST_ based on a dataset generated from pool‐seq exome capture for western larch populations (*n* = 40 individuals per population) that included over 1.4 million SNPs after filtering for missing values and minor allele frequency (B. Roskilly and B. Lind, unpublished data). *Q*_ST_ estimates higher than global *F*_ST_ were considered indicative of local adaptation for a given trait (Whitlock and Guillaume 2009).

### 2.7 Phenotypic Clines

We used simple linear regressions in R version 4.2.0 (R Development Core Team 2022) to test associations between BLUEs of the population phenotypes and selected environmental variables. Regression coefficients were considered significant at *p* < 0.05.

### 2.8 Comparison of Populations and Selected Full‐Sib Families Within Breeding Zones

Best linear unbiased estimates (BLUEs) were estimated for seed source type (natural population or full‐sib family) by breeding zone using the following mixed‐effects model:

(2) $$Y_{\text{ijkl}}=\mu +S_{j}+Z_{k}+{\left(S*Z\right)}_{\mathrm{jk}}+B_{l}+\epsilon_{\text{ijkl}},$$

where $Y_{\mathrm{ijk}}$ is the phenotype corresponding to individual *i* from seed source type *j*, breeding zone *k*, block *l*; μ is the phenotypic global mean across all individuals, *S* is the fixed effect of the *j*th seed source type, *Z* is the fixed effect of the *k*th breeding zone, S*Z the fixed effect of the seed source type by breeding zone interaction, *B* is the random effect of *l*th block, and $\epsilon_{\text{ijkl}}$ is the random residual error term including the spatial autocorrelation of individual seedling position. We tested for pairwise differences between BLUEs of seed source type phenotypes within breeding zones using pairwise *t*‐tests with the *asremlPlus* package for ASReml‐R version 4.0.

### 2.9 Trait Correlations

To test for correlations among traits, Pearson correlation coefficients were estimated with BLUEs of the population and family phenotypes. Pearson correlation coefficients were significant at *p* < 0.05.

### 2.10 Additive Genetic Variance and Heritability

We estimated additive genetic variance and narrow‐sense heritability in selected full‐sib families with the individual (animal) model approach using the following mixed‐effects model:

(3) $$Y_{\mathrm{ijk}}=\mu +B_{j}+G_{k}+\epsilon_{\mathrm{ijk}},$$

where $Y_{\mathrm{ijk}}$ is the phenotype corresponding to individual *i* from block *j* and individual additive genetic value *k*; μ is the phenotypic global mean across all individuals, *B* is the random effect of *j*th block, *G* is the random effect of the *k*th individual additive genetic value derived from the relationship matrix based on the pedigree of the full‐sib family structure, and $\epsilon_{\mathrm{ijk}}$ is the random residual error term.

Narrow‐sense heritability (*h* ^2^) for each trait was estimated as:

(4) $$h^{2}=\frac{\sigma_{a}^{2}}{\sigma_{p}^{2}},$$

where $\sigma_{a}^{2}$ is the additive genetic variance estimated from Equation (3) and $\sigma_{p}^{2}$ is the total phenotypic variance.

## 3 Results

### 3.1 Population Differentiation and Clines

The proportion of phenotypic variance among populations (*V*_POP_) was generally low but varied among traits, ranging from 0.02 to 0.16 (Table 1; Figure S3). The proportion of additive genetic variance among populations (*Q*_ST_) was low to moderate among traits, ranging from 0.10 to 0.28 (Table 1). *Q*_ST_ estimates were not strongly correlated with *V*_POP_ due to differences in additive genetic variation between traits.

**TABLE 1:** Population differentiation and variance components.

Trait	*σ* ^2^_p_	*σ* ^2^_e_	*V*_POP_	*Q*_ST_	Range
Bud flush (doy)	2.99	31.49	0.09	0.20	74–84
Early growth (cm)	1.72	22.02	0.07	0.28	13–22
Late growth (cm)	2.18	11.19	0.16	0.21	5–14
Annual growth (cm)	6.43	46.79	0.12	0.23	18–34
Final bud set (doy)	12.47	105.15	0.11	0.11	187–212
Cold injury (%)	1.65	96.52	0.02	0.10	43–59

*Note:* Phenotypic variance components: *σ* ^2^_p_ = phenotypic variance among populations, σ^2^_e_ = phenotypic variance within populations and the residual variance. Proportion of phenotypic variance among populations (*V*_POP_), additive genetic variance among populations (*Q*_ST_), and range of estimated population phenotypes (BLUEs). Bud flush and final bud set were analyzed as day of year (doy).

**FIGURE 2:**
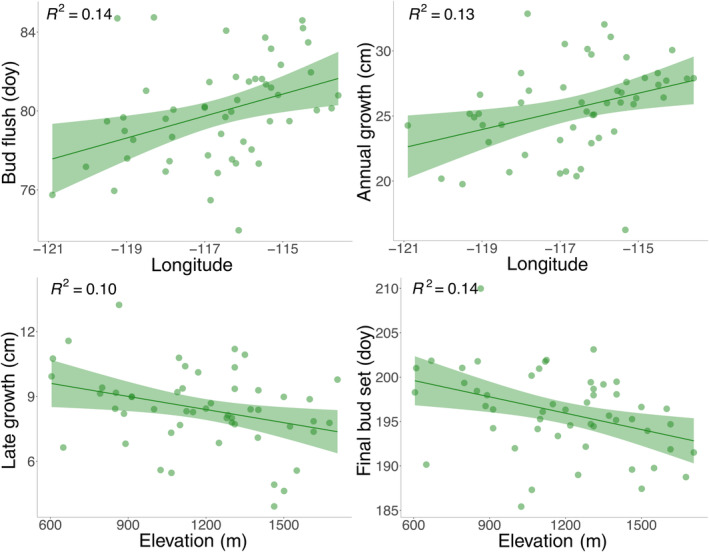
Regressions of bud break and longitude, annual height growth and longitude, late growth and elevation, bud set and elevation among populations (*n* = 52). Bud flush and final bud set were analyzed as day of year (doy). Shaded areas represent 95% confidence intervals and regressions are significant at a level of *p* < 0.05.

Population differentiation in annual growth was moderately low (*V*_POP_ = 0.12, *Q*_ST_ = 0.23; Table 1) and weakly associated with longitude (*R* ^2^ = 0.13, *p* = 0.01; Figure 2). The amount of early growth had a low *V*_POP_ estimate but moderate *Q*_ST_ value (*V*_POP_ = 0.07, *Q*_ST_ = 0.28; Table 1). Early growth was also weakly associated with longitude (*R* ^2^ = 0.15, *p* = 0.01; Table 2) and the length of the frost‐free period (*R* ^2^ = 0.09, *p* = 0.03; Table 2). Population differentiation for late growth was similar to annual growth (*V*_POP_ = 0.16, *Q*_ST_ = 0.21; Table 1). Late growth was associated with elevation (*R* ^2^ = 0.10, *p* = 0.01; Table 2) and latitude (*R* ^2^ = 0.08, *p* = 0.02; Table 2). Early and late growth were both highly correlated with annual growth among populations and selected full‐sib families (Figure S4).

**TABLE 2:** Coefficient of determination (*R* ^2^) and slope values for significant regressions between phenotypic traits and geographic or climatic variable of source populations at significance level of *p* < 0.05.

Trait	Environmental variable	*R* ^2^	Slope
Bud flush (doy)	Longitude (°)	0.14	0.56
Early growth (cm)	Longitude (°)	0.15	0.44
	FFP (days)	0.09	−0.03
Late growth (cm)	Elevation (m)	0.10	−0.002
	Latitude (°)	0.08	0.35
Annual growth (cm)	Longitude (°)	0.13	0.70
Final bud set (doy)	Elevation (°)	0.14	−0.006
	Latitude (°)	0.08	0.89

Abbreviation: FFP, frost‐free period length (days).

Population differentiation for timing of bud flush was low (*V*_POP_ = 0.09, *Q*_ST_ = 0.20; Table 1) and was weakly associated with longitude (*R* ^2^ = 0.14, *p* = 0.01; Figure 2). Bud flush timing was not correlated with annual growth or date of final bud set, either among populations or among selected full‐sib families (Figure S4). The timing of final bud set also had weak population differentiation (*V*_POP_ = 0.11, *Q*_ST_ = 0.11; Table 1). Final bud set was moderately correlated with annual growth among populations but not correlated among selected full‐sib families (Figure S4). Final bud set was weakly associated with elevation (*R* ^2^ = 0.14, *p* = 0.01; Table 2) and latitude (*R* ^2^ = 0.08, *p* = 0.05; Table 2).

Population differentiation for fall cold injury was very weak (*V*_POP_ = 0.02, *Q*_ST_ = 0.10; Table 1). Fall cold injury was not significantly associated with any climatic or geographic variable (Table S2). The range of values for population phenotypes was small, with only 16% average cold injury separating the least and most injured populations (Table 1, Figure S3).

### 3.2 Comparisons Between Natural Populations and Selected Full‐Sib Families

**FIGURE 3:**
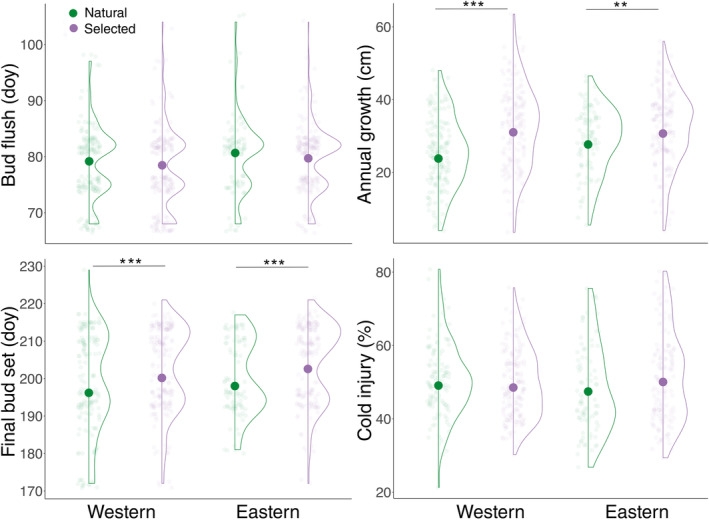
Distribution of individual data and estimated population and family phenotypes (bold points) for annual height growth, bud set and cold injury data compared between selected full‐sib families (*n* = 28) and natural populations (*n* = 23) from the eastern and western breeding zones of British Columbia. Significance of t‐tests: *p < 0.05, **p < 0.01, ***p < 0.001.

Selected full‐sib families from the breeding program averaged 21% greater annual growth than natural populations in both breeding zones (Figure 3). Annual growth had moderately low heritability (*h* ^2^ = 0.17; Table 3). Selected full‐sib families had greater early and late growth compared to natural populations (Figure S5). Late growth had moderate heritability (*h* ^2^ = 0.26) and early growth had low heritability (*h* ^2^ = 0.08; Table 3). Bud flush timing had low heritability (*h* ^2^ = 0.13; Table 3) and did not differ between selected full‐sib families and natural populations (Figure 3). Final bud set was delayed by 4.3 days on average in selected full‐sib families compared to natural populations in both breeding zones (Figure 3; Table S3) and had moderately high heritability (*h* ^2^ = 0.37; Table 3). Fall cold hardiness did not differ significantly between selected full‐sib families and natural populations (Figure 3) and had low heritability (*h* ^2^ = 0.07; Table 3).

**TABLE 3:** Trait heritability and variance components for selected full‐sib families.

Trait	*σ* ^2^_a_	*σ* ^2^_p_	*h* ^2^	se
Bud flush	5.87	43.87	0.13	0.08
Early growth	2.17	28.53	0.08	0.06
Late growth	4.03	15.46	0.26	0.10
Annual growth	10.94	65.10	0.17	0.09
Final bud set	49.53	135.34	0.37	0.12
Cold injury	7.09	106.31	0.07	0.06

*Note:* σ^2^_a_ = additive genetic variance estimated from pedigree, σ^2^_p_ = total phenotypic variance for the trait, narrow‐sense heritability (*h* ^2^), and standard error (se) for each phenotypic trait estimated from 28 full‐sib families from the breeding program of British Columbia.

## 4 Discussion

### 4.1 Weak Signals of Local Adaptation Among Natural Populations

We studied genetic variation in traits relevant to climate in natural populations and selectively bred families of western larch to better understand population differentiation, the quantitative genetics of climate‐relevant traits, and the implications for assisted migration and breeding for future climates. We found low differentiation among populations for all traits, with the proportion of phenotypic variance (*V*_POP_) ranging from 0.02 to 0.16. Estimates of the proportion of additive genetic variance due to population differences (*Q*_ST_) were not strongly correlated with *V*_POP_ due to differences in additive genetic variation among traits and ranged from 0.10 to 0.28 (Table 1). Although low, *Q*_ST_ estimates were higher than differentiation among populations for selectively neutral loci that had an estimated global *F*_ST_ = 0.01 (B. Roskilly and B. Lind, unpublished data), suggesting these traits are weakly locally adapted. Weak clines in height growth, bud flush, and bud set were found with longitude and elevation (Figure 2) but surprisingly not with the climatic variables for source populations that we expected to be drivers of local adaptation in temperate conifers (Table S2). We found that cold hardiness had the weakest population differentiation and had no significant clines. Comparisons between full‐sib families and natural populations indicate that selective breeding for faster growth has delayed bud set but maintained trait variation in bud flush phenology and cold hardiness. Collectively, our results indicate weak local adaptation in western larch and suggest phenotypic plasticity may be important in accommodating environmental variation across the species range (Leites and Benito Garzón 2023).

Population differentiation and clines in height growth typically reflect adaptation to growing season conditions (Alberto et al. 2013; Aitken and Bemmels 2016). In most temperate evergreen conifers, height growth tends to be faster in populations from warmer climates, reflected in clines with latitude, elevation or climate (Alberto et al. 2013). In this study, population differentiation for annual growth in western larch populations was low (Table 1), weakly correlated with longitude (Figure 2) and uncorrelated with climatic variables (Table S2). Comparisons of patterns in this study with the well‐studied species Douglas‐fir (*Pseudotsuga menziesii*) are informative as *Larix* and *Pseudotsuga* are sister genera in the Pinaceae with sympatric populations in their natural ranges. Population differentiation in height growth in western larch was lower in this study than for populations of Douglas‐fir (*V*_POP_ = 0.17; St Clair et al. 2005) and lodgepole pine (*Pinus contorta*; *V*_POP_ = 0.19; Liepe et al. 2016). Weak population differentiation in height growth for western larch was also found in a provenance trial in northern Idaho (Rehfeldt 1995) and in a series of provenance trials in southern British Columbia (Leites et al. 2012). Clines in height growth with elevation were detected in a northern Idaho provenance trial at 4 years (Rehfeldt 1995) and in 2‐year nursery bed trials (Rehfeldt 1982). Though we sampled a broad portion of the species range, it is possible that the less intensive distribution of sampled populations over topographically complex and climatically variable regions of the range or the warmer climate at the study site resulted in weaker associations with elevation than previous studies. These findings serve as an important reminder that signals of local adaptation found in common garden studies can be affected by the climatic variability represented in sampled populations and the test site climate.

As an early‐successional, shade‐intolerant species, indeterminate free growth is an important trait that enables western larch trees to take advantage of favorable current growing season conditions and gain a dominant or co‐dominant position in the canopy. Unlike most conifers in the Pinaceae, free growth extends beyond the juvenile stage and remains a significant component of growth potential in mature western larch trees (Schmidt and Shearer 1990). We could not separate determinate and free growth in this study because preformed and neoformed leaves were not readily distinguishable based on morphological differences, and so instead analyzed early and late growth. Early growth therefore includes both determinate growth from shoot primordia that overwintered in buds and indeterminate free growth from neoformed leaves and internodes during the growing season. Like annual growth, we found early growth had low population differentiation (Table 1) and weak clines with longitude and the length of the frost‐free period (Table 2). Faster early growth in eastern populations may reflect an adaptation to compensate for shorter growing seasons. Faster height growth rates in populations with shorter growing seasons have been found in other Pinaceae species, such as *Picea sitchensis* (Mimura and Aitken 2007).

Late growth included two forms of indeterminate growth: free growth and lammas growth. The latter results from reflushing after bud set in the same growing season. Late growth had weak clines with elevation and latitude following clines in final bud set (Table 2). Populations from lower elevations may have a greater capacity for lammas growth and later bud set to take advantage of favorable late‐season growing conditions such as those found at the study site. Earlier bud set in high elevation populations likely reflects adaptation to avoid early fall frost risks and shorter growing seasons. Late growth and bud set also had a weak negative relationship with latitude (Table 2), such that populations from higher latitudes had later bud set. While this was an unexpected pattern, given the limited variation in photoperiod over the latitudinal gradient sampled, it may be due to a moderate correlation between elevation and latitude of provenances (Figure S6), with higher latitude populations growing at lower elevations with less risk of early fall frost (Sang et al. 2021). It is also possible that the favorable late‐season growing conditions at the study site reduced population differentiation and weakened the strength of clines in final bud set by promoting reflushing after initial bud set. Expression of lammas growth at the study site was likely much greater than the frequency of lammas growth in their source environments (B. Jaquish, personal communication). However, our results are consistent with previous provenance trials that have generally found elevational clines of only modest slope in western larch, with populations on average needing to be separated by at least 400 m elevation to exhibit significant genetic differentiation (Rehfeldt and Jaquish 2010).

Deciduous tree species tend to break bud earlier than evergreen species because they need regrow their entire canopy to start photosynthesizing at the beginning of each growing season, which puts them at greater risk of late spring frosts (Panchen et al. 2014). We found that bud flush in western larch seedlings was on average 23 days earlier than interior variety Douglas‐fir seedlings from sympatric populations at the same common garden site in the same year (Nuhu 2022). Bud flush in our western larch seedlings occurred by March 21st, on average, nearly a month earlier than average bud flush in a previous provenance trial within the species range in northern Idaho (Rehfeldt 1992), suggesting that bud flush was substantially advanced by warmer temperatures at the study site. Bud flush was later in eastern populations following a weak cline with longitude (Figure 2). This result is not consistent with the general pattern found in evergreen conifers, where populations from colder or more continental climates typically have earlier bud flush due to lower chilling or heat sum requirements (Howe et al. 2003). Populations from the east may have higher chilling requirements that were met later at the warmer study site, tempering the advancement of bud flush. Though we found only a weak signal of local adaptation in bud flush, managers should be cautious of increasing risks of late spring frosts as a result of assisted migration and climate change given western larch's early bud flush phenology.

The weak population differentiation and lack of clines for cold hardiness in western larch are striking given that cold hardiness typically exhibits the strongest population differentiation and climatic clines of all seedling traits studied in temperate conifer species (Alberto et al. 2013; Leites and Benito Garzón 2023). Even though test temperatures resulted in optimal, intermediate levels of damage expected to maximize phenotypic variation using well established methods, only 16% difference in cold injury separated the least and most cold‐injured populations. This is consistent with the lack of significant population differentiation or clines for visible winter cold injury found in situ in a previous provenance trial at a substantially colder test site in northern Idaho (Rehfeldt 1995). Our results suggest western larch may be globally rather than locally adapted to cold temperatures. Even in the warm study site in mid‐October, we needed to test branch tissue samples at temperatures of −25 and −30°C to achieve targeted cold injury values of approximately 50%. These temperatures are well below the lowest average minimum temperatures for October at the five coldest source locations (−3.5°C) suggesting a wide safety margin in fall cold hardiness. Global adaptation to cold temperatures in western larch is consistent with the boreal evolutionary history of the *Larix* genus (LePage and Basinger 1995).

### 4.2 Selective Breeding for Faster Growth Delayed Bud Set With No Strong Effects on Bud Flush or Cold Hardiness

Selected full‐sib families had 21% greater annual height growth on average compared to natural populations (Figure 3), corroborating reported genetic gains in growth in a warmer test site located outside of established breeding zones (Forest Genetics Council of British Columbia 2021). Selection for greater annual growth has increased early and late growth in both breeding zones (Figure S5). Late growth had higher heritability than annual growth and early growth (Table 3). Early growth heritability may have been underestimated in part due to the inability to distinguish predetermined and free growth in study. On the other hand, estimated heritability of late growth in this study was comparable to a previous estimate based on 52 families from three natural populations in the Rocky Mountains (*h* ^2^ = 0.20; Rehfeldt 1992). Our heritability estimates for late growth and bud set timing are comparable to those for lammas growth and bud set timing, respectively, in Douglas‐fir (Kaya, Adams, and Campbell 1994; Howe et al. 2003). Late growth is increased through a delay of 4.3 days on average in final bud set in selected families compared to natural populations from both breeding zones (Figure 3; Table S3).

Selective breeding has had little impact on bud flush and fall cold hardiness in western larch (Figure 3). Selected families in the eastern zone had slightly elevated cold injury compared to natural populations but the difference was only marginally significant (*p* = 0.06) and the effect was very small (Figure 3). Cold hardiness and bud flush also had relatively low heritability (Table 3), lower than average cold hardiness heritability estimates for Douglas‐fir and lower than average estimates for bud flush heritability in other temperate conifer and broadleaf species (Howe et al. 2003). Consistent with these results, bud flush was not correlated with annual growth or final bud set in natural populations or among selected families (Figure S4), likely because indeterminate growth decouples bud flush from annual growth and bud set in western larch. We tested cold hardiness once over a 2‐week period in late October during a critical period of cold acclimation in the fall. It is possible that differences between seed sources may be greater at other time periods, for example, earlier in the fall or later in spring. Nonetheless, our findings suggest that selective breeding for faster growth has mostly maintained cold hardiness compared to natural populations, consistent with findings for lodgepole pine and interior spruce (hybrid populations of *Picea glauca*, *Picea engelmannii*, and *Picea sitchensis*; MacLachlan et al. 2017; MacLachlan, Yeaman, and Aitken 2018). However, genomic studies in lodgepole pine indicate that future responses to selection for growth may be constrained by correlated responses among growth, phenology and cold hardiness due to extensive pleiotropy (Maclachlan et al. 2021).

### 4.3 Potential Causes and Implications of Weak Local Adaptation in Western Larch

The weak signals of local adaptation to climate in western larch we found are consistent with previous work (Rehfeldt 1995; Rehfeldt and Jaquish 2010), though the clines found in our study may differ due to the warmer and milder climate of the study site or the distribution of sampled populations. High phenotypic variance within populations for traits in this study (Figure S3) as well as previous work suggest that local adaptation is not limited by low standing genetic variation (Rehfeldt 1992, 1995). Weak signals of local adaptation in this species are likely due to the relatively narrow climatic range inhabited by the species, which could be limited by ecological factors such as disturbance and succession (Rehfeldt and Jaquish 2010). Weak population structure due to population history and few geographic barriers to gene flow across the natural range may also contribute to weak signals of local adaptation. Neutral genetic differentiation among populations was notably low; *F*_ST_ = 0.01 based on over 1.4 million SNPs (B. Roskilly and B. Lind, unpublished data). This estimate is lower than that estimated with a similar set of SNPs for interior Douglas‐fir populations, which are sympatric with western larch populations but span a wider geographic and climatic range (*F*_ST_ = 0.03; Candido‐Ribeiro and Aitken 2024). Western larch also tends to be a minor component of the mixed‐conifer stands it inhabits, with smaller local population sizes (Schmidt and Shearer 1990). Low density stands may receive relatively high proportions of pollen originating from other locations which would contribute to low differentiation among populations.

This common garden experiment was conducted at a site outside the current natural species range to simulate the effects of warming at a sensitive early developmental stage. Short‐term common garden experiments can provide valuable insight into intraspecific genetic variation in climate‐relevant traits, facilitating in‐depth phenotyping not feasible in remote field locations, and can indicate potential drivers of clinal variation. However, to robustly test how far populations and species can be transferred to track climate change with assisted migration, populations and species should be tested in a wide range of climates in field reciprocal transplant studies (Wadgymar et al. 2022; Leites and Benito Garzón 2023). These studies should be monitored long term, as evidence of maladaptation can take decades to become apparent in long‐lived tree species (St. Clair et al. 2020) and could lead to more conservative climate‐based seed transfer guidelines (O'Neill et al. 2014). As drought becomes an increasingly dominant driver of tree mortality globally (Hartmann et al. 2022), intraspecific genetic variation and phenotypic plasticity of drought relevant traits also need to be better understood to inform assisted migration and breeding strategies (Moran et al. 2017; Candido‐Ribeiro and Aitken 2024).

The weak signals of local adaptation we found among western larch populations suggest limited benefits and risks of assisted gene flow among populations within the species range for mitigating maladaptation with climate change compared with many other tree species. Assisted migration of this species beyond the natural range is already underway based on previous research (Rehfeldt and Jaquish 2010; O'Neill et al. 2017). Our results also suggest that the selection of seed sources for planting in locations outside of the range will be less critical for western larch than for more locally adapted species. We hypothesize that western larch is a species that relies more on phenotypic plasticity than local adaptation to accommodate environmental variation. The ability of this species to respond to climate change may therefore be bounded by the climatic limits of phenotypic plasticity and its interplay with adaptation under changing climates.

## Conflicts of Interest

The authors declare no conflicts of interest.

## Supporting information


**Figure S1.** Distribution of mean temperature variables for population source climates and the common garden site.
**Figure S2.** Locations of 23 natural populations sampled from two breeding zones in British Columbia.
**Figure S3.** Distribution of trait variation within and among populations for annual growth, bud flush.
**Figure S4.** Heatmap of pairwise trait correlations among natural populations and full‐sib families.
**Figure S5.** Comparison of early and late growth between natural populations and selected full‐sib families.
**Figure S6.** Heatmap of pairwise trait Pearson correlation coefficients (*r)* among selected climate and geographic variables.

**Table S1.** Description of 52 natural populations sampled and their phenotypes.
**Table S2.** Regression coefficients (*R* ^2^) between phenotypic traits and selected geographic and climatic variables of the 52 natural populations.
**Table S3.** Phenotypes for natural populations and selected full‐sib families sampled from two breeding zones in British Columbia.

## Data Availability

The data used in this study are publicly available at the Dryad Digital Repository: https://doi.org/10.5061/dryad.h9w0vt4sb.

## References

[eva70001-bib-0001] Aitken, S. N. , and J. B. Bemmels . 2016. “Time to Get Moving: Assisted Gene Flow of Forest Trees.” Evolutionary Applications 9: 271–290. 27087852 10.1111/eva.12293 PMC4780373

[eva70001-bib-0002] Aitken, S. N. , and M. C. Whitlock . 2013. “Assisted Gene Flow to Facilitate Local Adaptation to Climate Change.” Annual Review of Ecology, Evolution, and Systematics 44: 367–388.

[eva70001-bib-0003] Alberto, F. J. , S. N. Aitken , R. Alía , et al. 2013. “Potential for Evolutionary Responses to Climate Change—Evidence From Tree Populations.” Global Change Biology 19: 1645–1661. 23505261 10.1111/gcb.12181 PMC3664019

[eva70001-bib-0004] Allen, C. D. , D. D. Breshears , and N. G. McDowell . 2015. “On Underestimation of Global Vulnerability to Tree Mortality and Forest Die‐Off From Hotter Drought in the Anthropocene.” Ecosphere 6: art129.

[eva70001-bib-0005] Bansal, S. , J. B. St. Clair , C. A. Harrington , and P. J. Gould . 2015. “Impact of Climate Change on Cold Hardiness of Douglas‐Fir (*Pseudotsuga menziesii*): Environmental and Genetic Considerations.” Global Change Biology 21: 3814–3826. 25920066 10.1111/gcb.12958

[eva70001-bib-0006] Bond, W. J. 1989. “The Tortoise and the Hare: Ecology of Angiosperm Dominance and Gymnosperm Persistence.” Biological Journal of the Linnean Society 36: 227–249.

[eva70001-bib-0007] Butler, D. G. , B. R. Cullis , A. R. Gilmour , B. J. Gogel , and R. Thompson . 2018. ASReml‐R Reference Manual Version 4. Vol. 176. Hemel Hempstead, UK: VSN International Ltd.

[eva70001-bib-0008] Candido‐Ribeiro, R. , and S. N. Aitken . 2024. “Weak Local Adaptation to Drought in Seedlings of a Widespread Conifer.” New Phytologist 241: 2395–2409. 38247230 10.1111/nph.19543

[eva70001-bib-0009] Erlichman, A. , L. Sandell , S. P. Otto , S. N. Aitken , and O. Ronce . 2024. “Planting Long‐Lived Trees in a Warming Climate: Theory Shows the Importance of Stage‐Dependent Climatic Tolerance.” Evolutionary Applications 17: e13711. 38894979 10.1111/eva.13711 PMC11183180

[eva70001-bib-0010] Flint, H. L. , B. R. Boyce , and D. J. Beattie . 1967. “Index of Injury—A Useful Expression of Freezing Injury to Plant Tissues as Determined by the Electrolytic Method.” Canadian Journal of Plant Science 47: 229–230.

[eva70001-bib-0011] Forest Genetics Council of British Columbia . 2021. Progress Report 2018–2021, edited by B. Barber . British Columbia, Canada: Forest Genetics Council of British Columbia.

[eva70001-bib-0012] Ghalambor, A. C. K. , J. K. Mckay , S. P. Carroll , et al. 2007. “Adaptive Versus Non‐Adaptive Phenotypic Plasticity and the Potential for Contemporary Adaptation in New Environments Published by: British Ecological Society Linked References Are Available on JSTOR for This Article: Adaptive Versus Non‐Adaptive Phenoty.” Functional Ecology 21: 394–407.

[eva70001-bib-0013] Hannerz, M. , S. N. Aitken , J. N. King , and S. Budge . 1999. “Effects of Genetic Selection for Growth on Frost Hardiness in Western Hemlock.” Canadian Journal of Forest Research 29: 509–516.

[eva70001-bib-0014] Hartmann, H. , A. Bastos , A. J. Das , et al. 2022. “Climate Change Risks to Global Forest Health: Emergence of Unexpected Events of Elevated Tree Mortality Worldwide.” Annual Review of Plant Biology 73: 673–702. 10.1146/annurev-arplant-102820-012804 35231182

[eva70001-bib-0016] Howe, G. T. , S. N. Aitken , D. B. Neale , K. D. Jermstad , N. C. Wheeler , and T. H. H. Chen . 2003. “From Genotype to Phenotype: Unraveling the Complexities of Cold Adaptation in Forest Trees.” Canadian Journal of Botany 81: 1247–1266.

[eva70001-bib-0045] Kaya, Z. , W. T. Adams , and R. K. Campbell . 1994. “Adaptive Significance of Intermittent Shoot Growth in Douglas‐Fir Seedlings.” Tree Physiology 14: 1277–1289. 10.1093/treephys/14.11.1277. 14967617

[eva70001-bib-0017] Leites, L. P. , and M. Benito Garzón . 2023. “Forest Tree Species Adaptation to Climate Across Biomes: Building on the Legacy of Ecological Genetics to Anticipate Responses to Climate Change.” Global Change Biology 29: 4711–4730. 37029765 10.1111/gcb.16711

[eva70001-bib-0018] Leites, L. P. , G. E. Rehfeldt , A. P. Robinson , N. L. Crookston , and B. Jaquish . 2012. “Possibilities and Limitations of Using Historic Provenance Tests to Infer Forest Species Growth Responses to Climate Change.” Natural Resource Modeling 25: 409–433.

[eva70001-bib-0019] LePage, B. A. , and J. F. Basinger . 1995. “The Evolutionary History of the Genus *Larix* (Pinaceae).” U.S. Department of Agriculture, Forest Service, Intermountain Research Station, General Technical Report GTR‐INT‐31: 19–29.

[eva70001-bib-0020] Liepe, K. J. , A. Hamann , P. Smets , C. R. Fitzpatrick , and S. N. Aitken . 2016. “Adaptation of Lodgepole Pine and Interior Spruce to Climate: Implications for Reforestation in a Warming World.” Evolutionary Applications 9: 409–419. 26834833 10.1111/eva.12345 PMC4721073

[eva70001-bib-0021] MacKenzie, W. H. , and C. R. Mahony . 2021. “An Ecological Approach to Climate Change‐Informed Tree Species Selection for Reforestation.” Forest Ecology and Management 481: 118705.

[eva70001-bib-0022] Maclachlan, I. R. , T. K. Mcdonald , B. M. Lind , L. H. Rieseberg , and S. Yeaman . 2021. “Genome‐Wide Shifts in Climate‐Related Variation Underpin Responses to Selective Breeding in a Widespread Conifer.” Proceedings of the National Academy of Sciences of the United States of America 118: e2016900118. 33649218 10.1073/pnas.2016900118 PMC7958292

[eva70001-bib-0023] MacLachlan, I. R. , T. Wang , A. Hamann , P. Smets , and S. N. Aitken . 2017. “Selective Breeding of Lodgepole Pine Increases Growth and Maintains Climatic Adaptation.” Forest Ecology and Management 391: 404–416.

[eva70001-bib-0024] MacLachlan, I. R. , S. Yeaman , and S. N. Aitken . 2018. “Growth Gains From Selective Breeding in a Spruce Hybrid Zone Do Not Compromise Local Adaptation to Climate.” Evolutionary Applications 13: 166–181. 10.1111/eva.12525 PMC5775489 29387153

[eva70001-bib-0025] Millar, C. I. , and N. L. Stephenson . 2015. “Temperate Forest Health in an Era of Emerging Megadisturbance.” Science 349: 823–826. 26293954 10.1126/science.aaa9933

[eva70001-bib-0026] Mimura, M. , and S. N. Aitken . 2007. “Adaptive Gradients and Isolation‐by‐Distance With Postglacial Migration in *Picea sitchensis* .” Heredity 99: 224–232. 17487214 10.1038/sj.hdy.6800987

[eva70001-bib-0027] Moran, E. V. , J. Lauder , C. Musser , A. Stathos , and M. Shu . 2017. “The Genetics of Drought Tolerance in Conifers.” New Phytologist 216: 1034–1048. 28895167 10.1111/nph.14774

[eva70001-bib-0028] Nuhu, J. 2022. Genetic Variation in Drought and Cold Tolerance in Selectively Bred and Natural Populations of Coastal Douglas‐Fir. Vancouver, BC: University of British Columbia.

[eva70001-bib-0029] O'Neill, G. A. , M. Stoehr , and B. Jaquish . 2014. “Quantifying Safe Seed Transfer Distance and Impacts of Tree Breeding on Adaptation.” Forest Ecology and Management 328: 122–130.

[eva70001-bib-0030] O'Neill, G. A. , T. Wang , N. Ukraintez , et al. 2017. A Proposed Climate‐Based Seed Transfer System for British Columbia. Victoria, BC: Crown Publications, Queen's Printer. Tech. Rep. 099.

[eva70001-bib-0031] Panchen, Z. A. , R. B. Primack , B. Nordt , et al. 2014. “Leaf Out Times of Temperate Woody Plants Are Related to Phylogeny, Deciduousness, Growth Habit and Wood Anatomy.” New Phytologist 203: 1208–1219. 24942252 10.1111/nph.12892

[eva70001-bib-0032] R Development Core Team . 2022. R: A Language and Environment for Statistical Computing. Vienna, Austria: R Foundation for Statistical Computing. http://www.r‐project.org.

[eva70001-bib-0033] Rehfeldt, G. E. 1982. “Differentiation of *Larix occidentalis* Populations From the Northern Rocky Mountains.” Silvae Genetica 31: 13–19.

[eva70001-bib-0034] Rehfeldt, G. E. 1992. “Breeding Strategies for *Larix occidentalis*: Adaptations to the Biotic and Abiotic Environment in Relation to Improving Growth.” Canadian Journal of Forest Research 22: 5–13.

[eva70001-bib-0035] Rehfeldt, G. 1995. “Genetic Variation, Climate Models and the Ecological Genetics of *Larix occidentalis* .” Forest Ecology and Management 78: 21–37.

[eva70001-bib-0036] Rehfeldt, G. E. , and B. C. Jaquish . 2010. “Ecological Impacts and Management Strategies for Western Larch in the Face of Climate‐Change.” Mitigation and Adaptation Strategies for Global Change 15: 283–306.

[eva70001-bib-0037] Sang, Z. , A. Hamann , and S. N. Aitken . 2021. “Assisted Migration Poleward Rather Than Upward in Elevation Minimizes Frost Risks in Plantations.” Climate Risk Management 34: 100380.

[eva70001-bib-0038] Schmidt, W. C. , and R. C. Shearer . 1990. “ *Larix occidentalis* Nutt., Western Larch. Silvics of North America. Volume 1 Conifers.” Agricultural Handbook 654: 227–237.

[eva70001-bib-0039] St. Clair, J. B. , G. T. Howe , and J. G. Kling . 2020. “The 1912 Douglas‐Fir Heredity Study: Long‐Term Effects of Climatic Transfer Distance on Growth and Survival.” Journal of Forestry 118: 1–13.

[eva70001-bib-0040] St Clair, J. B. , N. L. Mandel , and K. W. Vance‐Borland . 2005. “Genecology of Douglas Fir in Western Oregon and Washington.” Annals of Botany 96: 1199–1214. 16246849 10.1093/aob/mci278 PMC4247077

[eva70001-bib-0041] Valladares, F. , S. Matesanz , F. Guilhaumon , et al. 2014. “The Effects of Phenotypic Plasticity and Local Adaptation on Forecasts of Species Range Shifts Under Climate Change.” Ecology Letters 17: 1351–1364. 25205436 10.1111/ele.12348

[eva70001-bib-0042] Wadgymar, S. M. , M. L. DeMarche , E. B. Josephs , S. N. Sheth , and J. T. Anderson . 2022. “Local Adaptation: Causal Agents of Selection and Adaptive Trait Divergence.” Annual Review of Ecology, Evolution, and Systematics 53: 87–111. 10.1146/annurev-ecolsys-012722-035231 PMC10544833 37790997

[eva70001-bib-0043] Wang, T. , A. Hamann , D. Spittlehouse , and C. Carroll . 2016. “Locally Downscaled and Spatially Customizable Climate Data for Historical and Future Periods for North America.” PLoS One 11: 1–17. 10.1371/journal.pone.0156720 PMC4898765 27275583

[eva70001-bib-0044] Whitlock, M. C. , and F. Guillaume . 2009. “Testing for Spatially Divergent Selection: Comparing QST to FST.” Genetics 183: 1055–1063. 19687138 10.1534/genetics.108.099812 PMC2778959

