# Supplementary material for: Weak Local Adaptation to Climate in Seedlings of a Deciduous Conifer Suggests Limited Benefits and Risks of Assisted Gene Flow
Source: Evol Appl. 2024 Sep 15;17(9):e70001. doi: 10.1111/eva.70001 (PMC11403170; doi:10.1111/eva.70001)
Supplement: Supplementary file 1 — Figure S1. Distribution of mean temperature variables for population source climates and the common garden site. Figure S2. Locations of 23 natural populations sampled from two breeding zones in British Columbia. Figure S3. Distribution of trait variation within and among populations for annual growth, bud flush. Figure S4. Heatmap of pairwise trait correlations among natural populations and full‐sib families. Figure S5. Comparison of early and late growth between natural populations and selected full‐sib families. Figure S6. Heatmap of pairwise trait Pearson correlation coefficients (r) among selected climate and geographic variables. [file EVA-17-e70001-s002.docx]

**Figure S1.** Distribution of mean annual temperature (top), mean coldest month temperature (middle), and continentality (bottom) for the source climate of each natural population (light grey) over the 1961-1990 climate normal period and the common garden site (CG) for the years of the experiment (dark grey).

**Figure S2.** Locations of 23 natural populations sampled from the two breeding zones within the current natural range of western larch in British Columbia.

**Figure S3.** Distribution of variation within and among natural populations for annual growth, bud flush, final bud set, and cold injury. Individual values for each trait are shown in light green points and population phenotypes shown in dark green points.

**Figure S4**. Heatmap of pairwise trait Pearson correlation coefficients (*r)*. Upper triangle shows among-family phenotypes (BLUEs) and lower triangle is among-population phenotypes, in bold are correlation coefficients at significance level of *P* < 0.05.


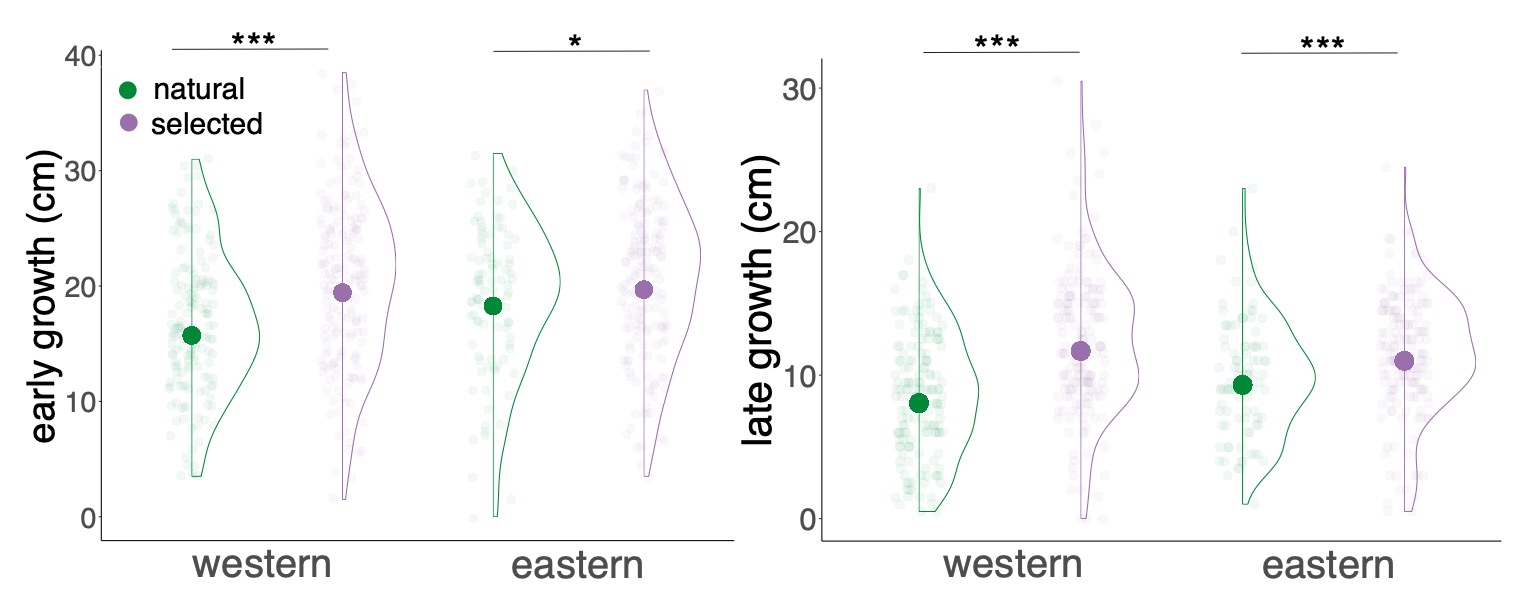


**Figure S5.** Distribution of individual data and estimated population and family phenotypes (bold points) for early growth and late growth between natural populations (*n*=23) and selected full-sib families (*n*=28) from the eastern and western breeding zones of British Columbia. **P*<0.05, ***P*<0.01, ****P*<0.001

**Figure S6.** Heatmap of pairwise trait Pearson correlation coefficients (*r)* among selected climate and geographic variables from 52 natural populations.
